# Alpha-synucleinopathy reduces NMNAT3 protein levels and neurite formation that can be rescued by targeting the NAD+ pathway

**DOI:** 10.1093/hmg/ddac077

**Published:** 2022-04-09

**Authors:** Richard B Parsons, Altin Kocinaj, Gustavo Ruiz Pulido, Sarah A Prendergast, Anna E Parsons, Paul D Facey, Frank Hirth

**Affiliations:** King’s College London, Institute of Pharmaceutical Science, 150 Stamford Street, London SE1 9NH, UK; King’s College London, Institute of Pharmaceutical Science, 150 Stamford Street, London SE1 9NH, UK; King’s College London, Institute of Pharmaceutical Science, 150 Stamford Street, London SE1 9NH, UK; King’s College London, Institute of Pharmaceutical Science, 150 Stamford Street, London SE1 9NH, UK; King’s College London, Institute of Pharmaceutical Science, 150 Stamford Street, London SE1 9NH, UK; Swansea University, Singleton Park Campus, Swansea University Medical School, Swansea SA2 8PP, UK; King’s College London, Institute of Psychiatry, Psychology and Neuroscience, Maurice Wohl Clinical Neurosciences Institute, Department of Basic & Clinical Neuroscience, 5 Cutcombe Road, London SE5 9RX, UK

## Abstract

Parkinson’s disease is characterized by the deposition of α-synuclein, which leads to synaptic dysfunction, the loss of neuronal connections and ultimately progressive neurodegeneration. Despite extensive research into Parkinson’s disease pathogenesis, the mechanisms underlying α-synuclein-mediated synaptopathy have remained elusive. Several lines of evidence suggest that altered nicotinamide adenine dinucleotide (NAD+) metabolism might be causally related to synucleinopathies, including Parkinson’s disease. NAD+ metabolism is central to the maintenance of synaptic structure and function. Its synthesis is mediated by nicotinamide mononucleotide adenylyltransferases (NMNATs), but their role in Parkinson’s disease is not known. Here we report significantly decreased levels of NMNAT3 protein in the caudate nucleus of patients who have died with Parkinson’s disease, which inversely correlated with the amount of monomeric α-synuclein. The detected alterations were specific and significant as the expression levels of NMNAT1, NMNAT2 and sterile alpha and TIR motif containing 1 (SARM1) were not significantly different in Parkinson’s disease patients compared to controls. To test the functional significance of these findings, we ectopically expressed wild-type α-synuclein in retinoic acid-differentiated dopaminergic SH-SY5Y cells that resulted in decreased levels of NMNAT3 protein plus a neurite pathology, which could be rescued by FK866, an inhibitor of nicotinamide phosphoribosyltransferase that acts as a key enzyme in the regulation of NAD+ synthesis. Our results establish, for the first time, NMNAT3 alterations in Parkinson’s disease and demonstrate in human cells that this phenotype together with neurite pathology is causally related to α-synucleinopathy. These findings identify alterations in the NAD+ biosynthetic pathway as a pathogenic mechanism underlying α-synuclein-mediated synaptopathy.

## Introduction

The onset and progression of Parkinson’s disease (PD) are characterized by synaptic dysfunction of dopaminergic (DA) neurones, leading to axonal degeneration and ultimately neuronal loss (1). The resulting loss of striatal dopamine gives rise to symptoms of resting tremor, bradykinesia, abnormal postural reflexes, rigidity and akinesia. The pathology of the disease is characterized by the deposition of α-synuclein (α-syn)-enriched Lewy bodies in remaining neurones (2). Under normal physiological conditions, α-syn functions in synaptic transmission and DA neurone physiology (3,4) where it acts as a chaperone in vesicle docking to active zones; however, upon aggregation, α-syn disrupts the active zone and impairs vesicular tethering and neurotransmitter recycling, resulting in subsequent synapse loss and neurodegeneration (5). As such, α-syn has been identified as a mediator of synaptopathy in PD (6–10).

Nicotinamide adenine dinucleotide (NAD+) is a central regulator of axon integrity and synaptic function (11) whose dysfunction has been implicated in neurodegeneration, including PD (12,13). NAD+ is produced *de novo* from nicotinamide by the sequential actions of nicotinamide phosphoribosyltransferase (NAMPT) and nicotinamide mononucleotide adenylyltransferase (NMNAT) (14,15). Mammals express three forms of NMNAT: 1, 2 and 3. NMNAT1 is primarily nuclear-localized, where it regulates the activities of NAD + -dependent enzymes such as poly(ADP-ribose) polymerase-1 and sirtuin-1 (16). NMNAT2 is enriched in membrane compartments including synaptic terminals and synaptic vesicles (17), where it regulates synapse homeostasis (18). Finally, NMNAT3 is expressed within mitochondria, where it regulates the provision of NAD+ for adenosine triphosphate (ATP) energy production (19). In addition to NMNAT function, the prodegenerative protein sterile alpha and TIR motif containing 1 (SARM1) can regulate NAD+ content via its NADase activity (15).

In addition to their enzymatic function of catalyzing NAD+ synthesis, NMNATs are also essential neuronal maintenance factors required for the integrity and function of nerve cells (18,20). Several studies have shown that transgenic expression of both NMNAT1 and NMNAT3 protect against axonal degeneration (21–24). NMNAT stabilizes T-bars that constitute the active zones of presynaptic terminals where vesicle fusion occurs, thereby mediating neurotransmitter release for neuronal communication (25). In *Drosophila*, dNMNAT interacts with bruchpilot, which together maintain the active zone and thus synaptic function (26,27).

Despite their importance in neuronal and axonal function, to date, no published studies have reported whether the expression of NMNAT proteins is disrupted in PD patient brain. Furthermore, it is unknown whether α-syn interacts with either of the three mammalian NMNAT proteins or whether NMNATs and SARM1 might be involved in α-syn-mediated pathology. In this study, we have compared the protein expression levels of all three NMNAT proteins and SARM1 in *post mortem* brain tissue of PD and nondisease control (NDC) subjects and correlated them with α-syn protein expression. Furthermore, we used the human neuroblastoma cell-line SH-SY5Y, ectopically expressing wild-type α-syn and terminally differentiated into DA neurones, to determine its effect upon the levels of NMNATs and SARM1 proteins and upon neurite and synapse formation *in vitro*. Finally, we determined the rescue potential of FK866 to mitigate the observed phenotypes caused by α-syn protein expression *in vitro.* Our results show that the expression level of NMNAT3 protein is decreased in PD compared to NDC subjects, which inversely correlates with the expression level of α-syn. Furthermore, we show that the ectopic expression of wild-type α-syn *in vitro* reduces NMNAT3 expression and induces a neurite pathology which can be rescued by targeting the NAD+ biosynthetic pathway using the NAMPT inhibitor, FK866.

## Results

### NMNAT3 protein level is decreased in the caudate nucleus but not cerebellum of Parkinson’s disease subjects

To determine whether the protein levels of enzymes of the NAD+ biosynthetic pathway are reduced in PD patient brain, we determined the amounts of NMNATs and SARM1 proteins in the caudate nucleus of NDC and PD subjects. We chose the caudate nucleus for these analyses because (i) all PD subjects in this study were of Braak stage 6 α-syn pathology (Table 1), and thus pathology would be present in the caudate nucleus (2,28), (ii) deficiencies in caudate nucleus function arising from dysconnectivity due to DA innervation loss are present in PD (29), and (iii) loss of DA projections into the caudate nucleus are involved in the pathophysiology of autonomic dysfunction in PD (30). From a structural perspective, we have previously demonstrated that there are significantly fewer neurones in the *substantia nigra* (SN) of PD subjects compared to NDCs for the patient cohort used for confocal microscopy studies in this study (31). As the caudate nucleus receives neuronal inputs from the SN (32) and PD motor symptom onset occurs when 80% of dopamine is lost in the caudate nucleus (33), any change in protein levels will result from the loss of DA inputs and not arise due to cell loss, which may potentially result in an artefactual decrease in protein levels in PD subjects, an effect we have observed in our previous studies (31). We therefore chose the caudate nucleus as a suitable brain region to study alterations in proteins levels related to PD. In support of this, we have previously shown that changes in protein levels and activity of nicotinamide *N*-methyltransferase within the caudate nucleus of PD compared to NDC subjects mirror those seen in other brain regions (31). Additionally, the levels of DA and its metabolites are reduced in PD patient caudate nucleus, mirroring what is observed in the SN (34). As a comparison and intrinsic control to caudate nucleus, we also investigated protein expression levels in the cerebellum. Cerebellum is commonly used as a control brain region in PD studies, with a recent meta-analysis showing that cerebellar involvement in PD pathology is limited (35), being functionally involved predominantly in compensatory effects upon the disease (36).

**Table 1:** Clinical data for quantitative western blot cohort

**Subjects**	**Sex**	**Age (years)**	**PMI** ^**a**^	**RIN** ^**b**^	**Age of disease onset**	**Disease duration** ^**c**^	**Neuropathological comorbidity**	**PD Braak stage**	**Genotype** ^**d**^	**Tau pathology**	**Dementia (age of onset, duration** ^**e**^ **)**
NDC1	F	71	17	5.8	–	–	Oedema	–	–	–	No
NDC2	F	93	22	3.9	–	–	Calcifications	–	–	–	No
NDC3	M	65	12	6.5	–	–	–	–	–	–	No
NDC4	F	78	23	7.5	–	–	–	–	–	–	No
NDC5	F	80	23	5.8	–	–	Alzheimer’s	–	–	-	No
NDC6	F	84	11	5.3	–	–	–	–	–	–	No
NDC7	M	82	48	6.8	–	–	–	–	–	–	No
NDC8	M	77	17	3.6	–	–	Alzheimer’s	–	–	–	No
NDC9	M	90	12	7.8	–	–	Mild aging and microvascular	–	–	–	No
NDC10	F	80	28	7.7	–	–	Alzheimer’s	–	–	–	No
NDC11	F	89	22	3.7	–	–	Aging and microvascular	–	–	–	No
NDC12	M	82	21	4.5	–	–	–	–	–	–	No
NDC13	M	88	22	5.4	–	–	–	–	–	–	No
NDC14	M	68	30	7.0	–	–	–	–	–	–	No
NDC15	M	84	5	7.0	–	–	–	–	–	–	No
NDC16	M	77	22	7.5	–	–	–	–	–	–	No
NDC17	M	68	10	7.5	–	–	Mild vascular	–	–	–	No
NDC18	M	85	23	6.0	–	–	Alzheimer’s	–	–	–	No
NDC19	M	68	24	7.5	–	–	Alzheimer’s	–	–	–	No
PD1	F	87	–	7.0	76	12	Alzheimer’s	6	LRRK2	2	Yes (1,86)
PD2	M	82	14	7.4	65	18	–	6	–	2	Y (4,78)
PD3	M	80	16	8.3	60	19	–	6	–	2	Yes (2,78)
PD4	M	73	19	7.4	65	9	–	6	–	<2	No
PD5	F	81	22	6.9	67	14	–	6	-	2	Yes (4,77)
PD6	M	82	10	6.0	72	11	–	6	–	2	Yes (5,77)
PD7	M	69	9	7.3	66	4	Alzheimer’s	6	–	2	No
PD8	M	74	20	6.2	50	25	Alzheimer’s	6	–	2	Yes (10,65)
PD9	F	76	22	6.9	62	11	Alzheimer’s	6	VPS35	2	Yes (7,70)
PD10	M	79	19	7.7	55	24	–	6	–	<2	No
PD11	M	65	14	7.3	60	6	–	6	–	<2	No
PD12	F	85	15	6.6	70	15	–	6	–	1	Yes (6,79)
PD13	M	75	3	6.8	65	10	–	6	–	2	No
PD14	F	84	19	6.9	66	18	Alzheimer’s	6	–	1	No
PD15	M	81	11	6.9	71	10	Alzheimer’s	6	–	2	Yes (3,78)
PD16	M	78	16	8.1	68	10	Alzheimer’s, Aβ pathology	6	EIF4G1	2	Yes (11,68)
PD17	M	84	21	7.0	79	6	Alzheimer’s	6	–	2	No
PD18	M	82	12	7.6	64	18	Alzheimer’s	6	–	1	Yes (2,80)
PD19	M	74	17	3.9	58	16	Alzheimer’s	6	–	1	Yes (5,70)

^a^Post-mortem interval (PMI) is given in hours.

^b^RNA integrity number (RIN) is used as an indicator of agonal state and thus tissue quality.

^c^Disease duration is given in years.

^d^The genotype for PD-related genes was determined by the Parkinson's UK Brain Bank: LRRK2, leucine rich repeat kinase-2; VPS35, vacuolar protein sorting-associated protein 32 homolog 1; EIF4G1, eukaryotic translation initiation factor 4 gamma 1.

^e^Age of dementia onset and duration are given in years.

**Figure 1:**
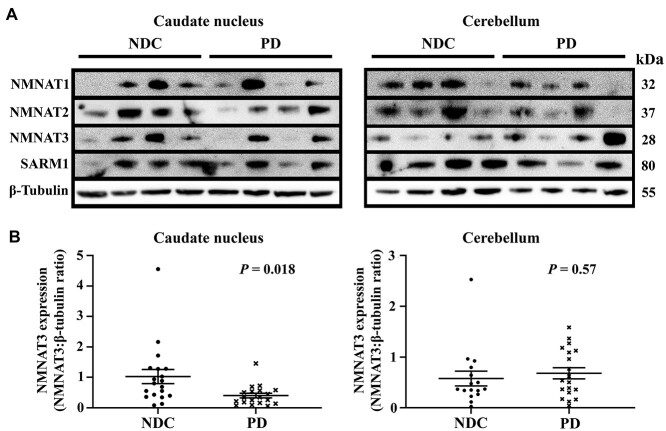
Decreased expression of NMNAT3 protein in the caudate nucleus of Parkinson’s disease patient brain. (**A**) Representative western blot of four non-disease control and four Parkinson’s disease subjects. Caudate nucleus and cerebellum samples were lysed in RIPA buffer and NMNATs −1, −2, −3, SARM1 and β-tubulin loading control were subsequently detected with specific antibodies. Bands were visualized using electrochemiluminescence detection. (**B**) Quantification of NMNAT3 protein expression. NMNAT3 band intensities were quantified by densitometric analysis using FIJI ImageJ v.1.53a and were normalized for β-tubulin. Results were expressed as ratio ± SEM (*n* = 19 for non-disease controls, *n* = 19 for Parkinson’s disease subjects). Statistical analysis comprised Student’s *t*-test with Welch correction and was performed using Prism v8.3. Abbreviations: NDC, non-disease control subjects; PD, Parkinson’s disease subjects; NMNAT, nicotinamide mononucleotide adenylyltransferase; SARM1, sterile alpha and TIR motif containing 1.

NMNAT1–3 and SARM1 proteins were detected as proteins of 32, 37, 28 and 80 kDa respectively, in NDC and PD subjects in both brain regions investigated (Fig. 1A). Levels of protein expression for all proteins varied among subjects in both groups. The protein expression level of NMNAT3 (Fig. 1B) was significantly decreased in the caudate nucleus of PD compared to NDC subjects (1.03 ± 0.23 versus 0.4 ± 0.08, *P* = 0.02) but was not significantly different in the cerebella of NDC compared to PD subjects (0.58 ± 0.15 versus 0.68 ± 0.11, *P* = 0.57). The protein expression levels of NMNAT1 and 2 and SARM1 were not significantly different in either brain region of PD compared to NDC subjects (Supplementary Material, Fig. S1 and Supplementary Material, Table S1).

Next, we determined whether there were any clinicopathological confounding factors which influenced the amount of NMNAT3 protein in the caudate nucleus. NMNAT3 protein levels were significantly higher in PD subjects with dementia than those without (0.5 ± 0.11 versus 0.19 ± 0.04, *P* = 0.02, *n* = 12 and 6, respectively). In contrast, NMNAT3 protein levels in PD subjects were independent of both Tau score (Supplementary Material, Table S2) and Alzheimer’s disease (AD) pathology (Supplementary Material, Table S3). There was no correlation between NMNAT3 expression levels and age at death, *post mortem* interval or RNA integrity number either in PD subjects or for the cohort as a whole (Supplementary Material, Table S4). Likewise, for PD subjects, there was no correlation between NMNAT3 protein expression levels and age of disease onset, disease duration, and age of onset of, or duration of, dementia (Supplementary Material, Table S4). Together these data identify significantly decreased levels of NMNAT3 protein expression in caudate nucleus but not cerebellum of PD patient brain.

### NMNAT3 and α-synuclein protein levels were inversely correlated in caudate nucleus of Parkinson’s disease subject brain

Previous studies showed that increased levels of α-syn protein directly correlate with time of disease onset and severity, including autosomal dominant forms of PD caused by duplication and triplication of the *SNCA* locus encoding α-syn (37,38). We therefore investigated whether the levels of α-syn protein may correlate with decreased level of NMNAT3 protein in the caudate nucleus of these PD subjects compared to NDC subjects.

**Figure 2:**
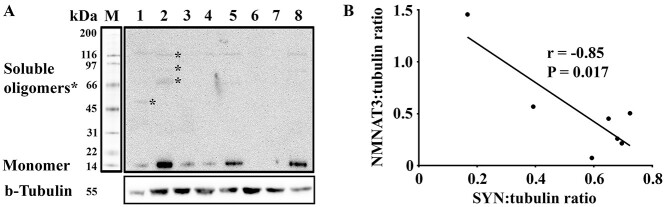
Inverse correlation of NMNAT3 and monomeric α-syn expression levels in caudate nucleus of Parkinson’s disease brain. (**A**) Western blot detection of α-syn in caudate nucleus RIPA lysates of Parkinson’s disease patients. α-syn was detected using anti-α-syn primary antibody and bands were visualized using electrochemiluminescence detection. Soluble oligomers of α-syn are identified by ^*^. (**B**) Correlation between tubulin-normalized NMNAT3 and monomeric α-syn expressions was performed by the Spearman correlation coefficient analysis using Prism v.8.3. For all panels: 1–8 = Parkinson’s disease sample. Abbreviations: NMNAT3, nicotinamide mononucleotide adenylyltransferase 3; SYN, α-synuclein; kDa, kilodaltons; M, molecular weight markers; 1–8, sample lanes.

In all brain samples investigated, α-syn was detected as multiple bands (Fig. 2A), corresponding to various soluble aggregates of α-syn (Table 2). Only 7 of the tested PD subjects expressed a protein of approximately 15 kDa corresponding to monomeric α-syn. We focused our analysis on the quantification of this 15 kDa band and correlated its expression level with those of NMNAT3 protein in these seven subjects. As a result, we found that increased levels of monomeric α-syn protein correlated with decreased levels of NMNAT3 protein (Fig. 2B, *r* = −0.85, *P* = 0.017). Correlation analysis of α-syn and NMNAT3 protein expressions using NDC subjects, either separately or pooled with the PD patient cases, revealed no correlation between α-syn and NMNAT3 protein levels (*r* = 0.44, *P* = 0.56, *n* = 6 for NDC subjects; *r* = −0.69, *P* = 0.84, *n* = 13 for whole cohort). We did not quantify the levels of α-syn oligomers and aggregates due to the difficulty in reliably isolating and quantifying them using quantitative western blotting. Together these data identify an inverse correlation between α-syn and NMNAT3 protein levels in the caudate nucleus of PD patient brain.

### NMNAT3 colocalization with DA neurones of the SN pars compacta was decreased in PD subject brain

**Figure 3:**
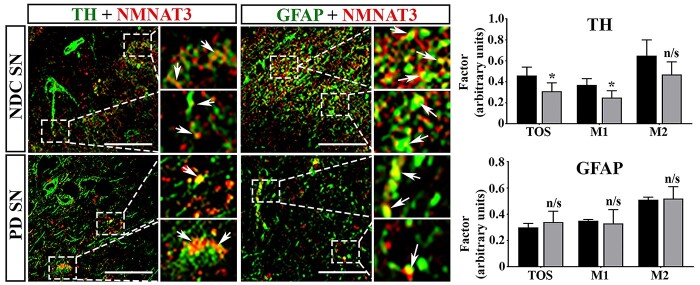
The colocalization of NMNAT3 and TH was decreased in the SN of Parkinson’s disease subjects. The localizations of NMNAT3, TH and GFAP were imaged using dual-label confocal microscopy. Target proteins were detected using a combination of specific primary and fluorescent secondary antibodies, with TH and GFAP imaged using AlexaFluor488 and NMNAT3 imaged using AlexaFluor594. Areas of interest are magnified. Arrow heads indicate areas of colocalization. Scale bar = 50 μm. Colocalization between NMNAT3 and both TH and GFAP was assessed using the EzColocalization plug-in v1.1.3 in FIJI Image-J v1.53a and expressed as TOS and Mander’s coefficients, using Coste’s thresholds. For all panels: TH, tyrosine hydroxylase; GFAP, glial fibrillary acidic protein; NMNAT3, nicotinamide mononucleotide adenylyltransferase3; SN, *substantia nigra*; NCD, non-disease controls; PD, Parkinson’s disease; TOS, threshold overlap score; M1 and M2, Mander’s coefficients; ^*^ = *P* < 0.05; n/s, not significant.

Although NMNAT3 expression in the caudate nucleus of PD subjects was significantly lower than NDC subjects, our next step was to confirm that this was reproduced in the SN. Due to the significant loss of DA neurones in the SN in this cohort, quantitative western blotting is not an appropriate method for this analysis. Instead, we assessed the expression of NMNAT3 in NDC and PD SN using confocal microscopy and quantitatively compared the degree of colocalization between tyrosine hydroxylase (TH)-positive neurones and NMNAT3, using colocalization with glial acidic fibrillary protein (GFAP)-positive glia as a control (Fig. 3). Colocalization correlation between NMNAT3 and each cell-type marker was assessed using threshold overlap scores (TOS) (39). The percentage overlap between the expression of each protein was estimated using Mander’s colocalization coefficients M1 and M2, with M1 measuring the percentage overlap between NMNAT3 and cellular markers and M2 measuring the percentage overlap between cellular markers and NMNAT3 (40). The advantage of this approach is that it analyses target protein levels in a desired cell type even when the numbers of that cell-type are low due to extensive degeneration of a brain region, thus overcoming the limitation of quantitative western blotting. Hence, we have used a change in colocalization with a phenotypic marker as a measure of changes in protein expression between the two subject groups (Fig. 3).

**Table 2:** Summary of soluble aggregates of α-synuclein detected in caudate nucleus of patient cohort using western blotting

**Calculated molecular weight (kDa)** ^**a**^	**Expected molecular weight (kDa)**	**Species**
15	14	Monomer
52	42	Trimer
62	56	Tetramer
91	84	Hexamer
118	112	Octamer

^a^Molecular weight was estimated using a calibration line constructed using PrecisionPlus™ protein molecular weight markers.

In both NDC and PD subject SN, NMNAT3 colocalized with TH within DA neurones. In NDC subjects, NMNAT3 expression was present predominantly within axons of DA neurones, whereas in PD subjects NMNAT3 expression was predominantly in cell bodies. NMNAT3 was expressed in glial cells of both NDC and PD subjects.

Calculation of TOS for DA expression revealed that colocalization between NMNAT3 and TH was not complete and was lower in PD compared to NDC subjects (0.46 ± 0.08 versus 0.31 ± 0.08, *P* = 0.037, *n* = 4 for each). Calculation of M1 revealed a significant reduction in the proportion of NMNAT3 expressed in TH-positive DA neurones in PD SN (37 ± 6% versus 25 ± 7%, *P* = 0.037, *n* = 4 for each), whereas M2 revealed no change in the proportion of TH expressed in NMNAT3-positive neurones (65 ± 15% versus 47 ± 12%, *P* = 0.11, *n* = 4 for each), suggesting that those TH-positive DA neurones remaining still expressed NMNAT3. Calculation of TOS, M1 and M2 for glial expression revealed that these parameters did not alter in NDC and PD subjects (TOS: 0.3 ± 0.03 versus 0.34 ± 0.083, *P* = 0.4; M1: 35 ± 1% versus 33 ± 11%, *P* = 0.72; M2: 51 ± 2% versus 52 ± 9%, *P* = 0.84; *n* = 4 per group for each). Taken together, these results demonstrate a reduction in NMNAT3 colocalization within TH-positive DA neurones but not glial cells in the SN of PD subjects.

### The expression of wild-type α-synuclein causes decreased levels of NMNAT3 protein *in vitro*

Having demonstrated that NMNAT3 protein expression was inversely correlated with α-syn protein expression, our next step was to determine whether PD-related α-syn expression functionally correlates with NMNAT3 protein expression levels. To test this hypothesis, we turned to an established *in vitro* model of PD, the differentiated SH-SY5Y DA cell model (41,42). In addition to their human origin, SH-SY5Y cells are a pan-neuronal cell line which can be differentiated into DA neurone-like cells in response to low serum and retinoic acid treatment. Although differentiation using this combination can result in a partially differentiated DA phenotype, this is associated with short (~3 days) application (43). We have consistently shown that differentiation using 10 μM retinoic acid for 7 days results in a terminally differentiated DA phenotype as evidenced by (i) the increased expressions of the neuronal marker neuronal-specific enolase and the post-mitotic marker Neuronal Nuclear protein/FOX-3 (NeuN); (ii) cell cycle arrest and cessation of proliferation; (iii) a change from an epithelial to a neuronal morphology with a concomitant increase in neurite density; and (iv) increased levels of the DA markers dopamine D2 receptor, TH and the vesicular monoamine transporter 1 (41,43,44). Alternative methods for differentiation have been reported, including the combination of retinoic acid and brain-derived growth factor (42); however, we have recently demonstrated that this combination results in the production of a robust cholinergic phenotype, as evidenced by increased expression and cellular activities of acetylcholinesterase and choline acetyltransferase, an increase in the expression of the cholinergic receptor genes *CHRNA4, CHRNA6, CHRM3* and *CHRM4*, and an increase in the expression of beta-site APP-cleaving enzyme 2 (*BACE2*), microtubule protein Tau (*MAPT*) and ADAM metallopeptidase domain 10 (*ADAM10*), all genes linked with the AD cascade (45). SH-SY5Y do not express appreciable levels of endogenous α-syn (46–48), making them an ideal PD-related *in vitro* system to study the effects of expression of α-syn. We therefore used SH-SY5Y cells and differentiated them into DA neurones to investigate the effects of ectopic α-syn expression and its potential impact on NMNAT3 protein expression. We used an established SH-SY5Y cell-line which stably expresses human wild-type α-syn *N*-terminally fused to enhanced green fluorescent protein (EGFP) (SH-SY5Y^WT^) as previously described (49), thus mimicking autosomal dominant forms of PD caused by multiplication of the α-syn-encoding *SNCA* locus (37,38). As a control, further cells were stably transfected with empty vector expressing EGFP alone (SH-SY5Y^MOCK^).

**Figure 4:**
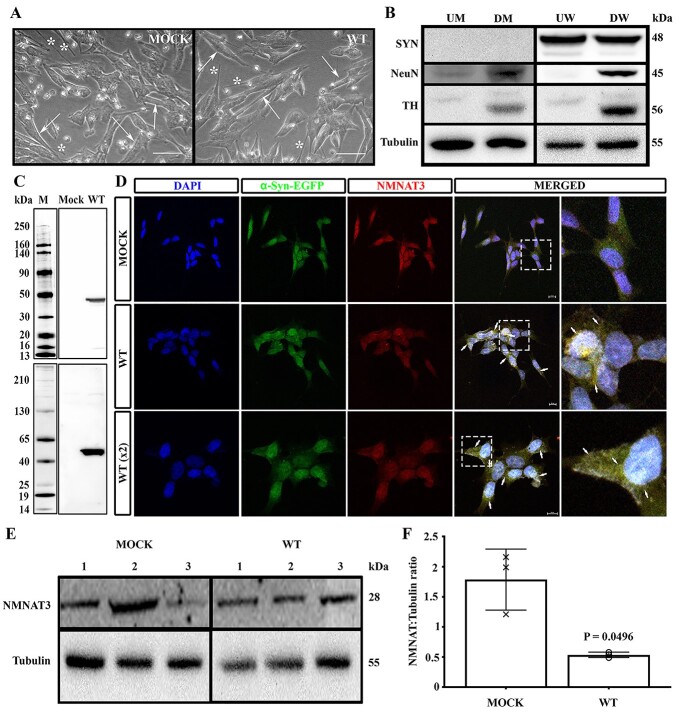
The expression of wild-type α-SYN decreased NMNAT3 protein expression in differentiated, DA SH-SY5Y cells. Cells stably expressing green fluorescent protein or wild-type α-SYN *N*-terminally fused to EGFP were differentiated with 10 μM retinoic acid for 7 days. (**A**) Cells were imaged using an Axiovert C40 microscope under phase-contrast illumination. Arrows indicate cell bodies; ^*^ indicates neurite projections. Scale bar = 50 μm. (**B**) Cells were lysed using RIPA and the expressions of α-SYN, the neuronal marker NeuN, TH and β-tubulin proteins were detected using specific antibodies by western blotting. Bands were visualized using electrochemiluminescence detection and quantified using FIJI ImageJ v.1.53a. (**C**) α-syn aggregates were assessed using an antibody raised against monomeric α-syn (top image) and an antibody raised against aggregate forms of α-syn (bottom image) using western blotting. (**D**) The localizations of EGFP, α-SYN-EGFP and NMNAT3 were imaged using confocal microscopy, with EGFP and α-SYN-EGFP imaged using facile fluorescence and NMNAT3 imaged using a combination of mouse-anti-NMNAT3 and chicken–anti-mouse AlexaFluor594. Nuclei were counterstained using DAPI. Cells were imaged as Z-stacks and shown as maximum projection images. Areas of interest are magnified. Arrows show examples of colocalization between α-SYN-EGFP and NMNAT3. All images were imaged using a x40 objective lens, with WT (x2) panels imaged at 2-fold magnification using a x40 lens. Scale bar = 10 μm. (**E**) NMNAT3 protein expression was detected in cell RIPA lysates by western blotting using an anti-NMNAT3 primary antibody. Bands were visualized using electrochemiluminescence detection. (**F**) Quantification of NMNAT3 protein expressions in cell RIPA lysates was performed by densitometric analysis using FIJI ImageJ v.1.53a, normalized for β-tubulin expression and expressed as ratio ± SD (*n* = 3). Statistical analysis comprised Student’s *t*-test with the Welch correction using Prism v.8.3. For all panels: MOCK, SH-SY5Y transfected with empty vector; WT, SH-SY5Y expressing wild-type α-SYN; UM, undifferentiated parental SH-SY5Y cells; DM, differentiated parental SH-SY5Y cells; UW, undifferentiated wild-type α-SYN-expressing cells; DW, differentiated SH-SY5Y wild-type α-SYN-expressing cells; M, molecular weight markers; kDa, kilodaltons. Abbreviations: SYN, synuclein; TH, tyrosine hydroxylase; NMNAT3, nicotinamide mononucleotide adenylyltransferase 3.

Upon differentiation with retinoic acid, both SH-SY5Y^MOCK^ and SH-SY5Y^WT^ demonstrated a neuronal phenotype as evidenced by elongated cell bodies and the production of neurite processes (Fig. 4A). Terminal differentiation in both cell lines was confirmed by the detection of the post-mitotic neuronal marker NeuN (50,51) as a protein of approximately 45 kDa, the intensity of which was increased in both differentiated SH-SY5Y^MOCK^ and SH-SY5Y^WT^ cells (Fig. 4B). Differentiation into a DA phenotype was confirmed by the detection of TH as a protein of approximately 56 kDa, the intensity of which was increased upon differentiation in both cell lines (Fig. 4B).

As expected, endogenous α-syn protein was not detected in SH-SY5Y^MOCK^ either before or after differentiation. Wild-type α-syn protein was expressed in both undifferentiated and differentiated SH-SY5Y^WT^ cells as a recombinant protein of approximately 48 kDa consistent with monomeric α-syn *N*-terminally fused to EGFP, which is in accord with previous reports for this plasmid construct (49) (Fig. 4B). α-Syn-EGFP was solely monomeric in proteins isolated from differentiated SH-SY5Y^WT^ cells, which was confirmed using an antibody raised against aggregated forms of α-syn (Fig. 4C). In SH-SY5Y^WT^, α-syn-EGFP was expressed throughout the cell body (Fig. 4D). There was significant colocalization between α-syn-EGFP and NMNAT3, which was not evident between EGFP and NMNAT3 in SH-SY5Y^MOCK^.

NMNAT3 was expressed as a protein of 28 kDa in both SH-SY5Y^MOCK^ and SH-SY5Y^WT^ cells (Fig. 4E). Quantitative assessment of NMNAT3 protein expression revealed its reduction by 70% in differentiated SH-SY5Y^WT^ when compared to SH-SY5Y^MOCK^ cells (1.787 ± 0.51 versus 0.54 ± 0.05, *P* = 0.0496, *n* = 3 for both) (Fig. 4F). To rule out this phenotype was caused by impaired *NMNAT3* gene expression or transcription, we carried out quantitative polymerase chain reaction (qPCR), which revealed there was no significant reduction in NMNAT3 mRNA level in SH-SY5Y^WT^ (0.91 ± 0.11 versus 1.11 ± 0.14, *P* = 0.104, *n* = 3). Together these data suggest that ectopic expression of wild-type monomeric α-syn causes a decrease in the amount of NMNAT3 protein in differentiated, DA neurone-like SH-SY5Y cells.

### The expression of α-synuclein reduced neurite number but not their average length

Accumulation of α-syn has been hypothesized to be a cause of PD-related synaptopathy, leading to the deconstruction of neurites and synapses and the subsequent degeneration of nerve cells (5,52). We therefore investigated whether, in addition to its impact on NMNAT3 protein levels, α-syn also affected neurite length and number.

**Figure 5:**
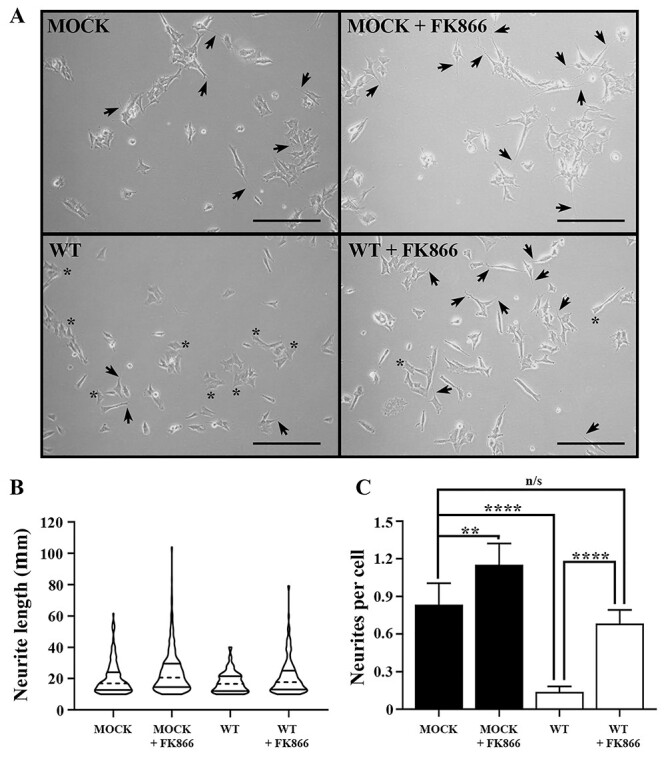
Treatment with the nicotinamide phosphoribosyltransferase inhibitor FK866 rescues α-SYN-mediated neurite pathology in differentiated, DA SH-SY5Y cells. (**A**) Cells were cultured for 24 hours with media supplemented with vehicle-only or 50 nM FK866, after which images were captured using an Axiovert C40 microscope under phase contrast illumination. (**B**) Neurite length was measured using the NeuronJ plugin v.1.4.3 for FIJI ImageJ v.1.53a. Neurites of less than 10 μm were not considered as true neurites and thus omitted from all calculations. Neurite length was plotted as μm, median (dashed line) and interquartile range (bold lines) using violin plots. (**C**) The number of neurites per cell were calculated and expressed as ± SD (*n* = 240 cells for parental SH-SY5Y, 366 for parental SH-SY5Y + FK866, 332 for wild-type α-SYN-expressing SH-SY5Y, 336 for wild-type α-SYN-expressing SH-SY5Y + FK866). Statistical analysis comprised QQ plots of residuals followed by parametric one-way ANOVA with Tukey’s multiple comparisons tests using Prism v.8.3. For all panels: MOCK = SH-SY5Y cells expressing EGFP; WT = SH-SY5Y cells stably expressing wild-type α-SYN *N*-terminally fused to enhanced green fluorescence protein; arrows = neurites; ^*^ = axonal horn; n/s, not significant; ^*^^*^ = *P* < 0.01; ^*^^*^^*^ = *P* < 0.001; ^*^^*^^*^^*^ = *P* < 0.0001. Scale bar = 100 μm.

In accord with Figure 4A, most differentiated SH-SY5Y^MOCK^ cells possessed neurite processes (Fig. 5A). In contrast, differentiated SH-SY5Y^WT^ cells expressing α-syn had significantly fewer neurites, with many cells possessing only the axonal cone. We quantified these changes by counting the number of neurites per cell and the length of detectable neurites (Fig. 5B). Violin plots revealed that most neurites were relatively short for both cell lines. There were more neurites of longer length in differentiated SH-SY5Y^MOCK^ compared to SH-SY5Y^WT^ cells, with the maximal length almost twice as long. However, the average length of SH-SY5Y^MOCK^ neurites was not significantly different compared to SH-SY5Y^WT^ (19.97 ± 10.09 versus 17.58 ± 6.63 μm, *P* > 0.9999).

In contrast, differentiated SH-SY5Y^WT^ cells had significantly fewer neurites per cell, with many neurones demonstrating only the axonal cone with no associated neurite (Fig. 5A). The expression of wild-type α-syn protein in differentiated SH-SY5Y^WT^ cells significantly decreased the number of neurites per cell compared to SH-SY5Y^MOCK^ cells (0.84 ± 0.17 versus 0.14 ± 0.17, *P* < 0.0001) (Fig. 5C). Together these data suggest that expression of α-syn decreases neurite number but not their average length in differentiated, DA neurone-like SH-SY5Y cells.

### α-Synuclein-mediated neurite pathology is rescued by the NAMPT inhibitor FK866

Several lines of evidence suggest α-syn-mediated synaptopathy and subsequent neurodegeneration might be related to altered NAD+ metabolism, a process that also involves NMNAT3 function (12). Since the expression of α-syn altered NMNAT3 protein levels (Fig. 4) and decreased neurite numbers (Fig. 5) in our cell model, we wondered whether pharmacological targeting of NAD+ biosynthesis might be able to mitigate or even rescue some of the observed phenotypes. We therefore applied the NAMPT inhibitor FK866 which was previously shown to rescue SARM1-dependent axonal degeneration (53).

Upon incubation with 50 nM FK866 for 24 hours, both SH-SY5Y^MOCK^ and SH-SY5Y^WT^ cells demonstrated a change in morphology (Fig. 5A), with each showing an apparent increase in the number of neurites and most axonal cones having an associated neurite. The distribution of neurite length in both cell lines shifted towards neurites of longer lengths; however, this was only significant for differentiated SH-SY5Y^MOCK^ cells incubated with FK866 (19.97 ± 10.09 versus 24.26 ± 13.32 μm, *P* < 0.0001), with no significant increase for differentiated SH-SY5Y^WT^ cells incubated with FK866 (17.58 ± 6.63 versus 21.1 ± 11.46 μm, *P* = 0.58) (Fig. 5B). There was no significant difference between neurite lengths of SH-SY5Y^MOCK^ and SH-SY5Y^WT^ cells incubated with FK866 (*P* > 0.9999) (Fig. 5B).

Furthermore, incubation with FK866 increased the average number of neurites per cell for both cell lines (0.84 ± 0.17 versus 1.15 ± 0.17, *P* = 0.008 for SH-SY5Y^MOCK^, 0.14 ± 0.17 versus 0.69 ± 0.11, *P* < 0.0001 for SH-SY5Y^WT^) (Fig. 5C). Remarkably, incubating differentiated SH-SY5Y^WT^ cells with FK866 returned the number of neurites per cell to that observed for untreated SH-SY5Y^MOCK^ cells (0.84 ± 0.17 versus 0.69 ± 0.11, *P* = 0.31) (Fig. 5C). Together these data suggest that the NAMPT inhibitor FK866 can rescue neurite pathology caused by α-syn expression in differentiated, DA neurone-like SH-SY5Y cells.

## Discussion

Our results presented here identify decreased levels of NMNAT3 protein in the caudate nucleus and in axonal projections of DA neurones of the SN in subjects who have died with PD. In PD patient samples, the decrease in NMNAT3 protein expression levels was found to be inversely correlated with levels of monomeric α-syn. Ectopic expression of wild-type monomeric α-syn in human cells resulted in decreased levels of NMNAT3 protein, corresponding to the situation detected in caudate nucleus of PD subject brain. Wild-type monomeric α-syn expression caused neurite pathology which could be rescued by FK866, a non-competitive inhibitor of NAMPT that acts as a key enzyme in the regulation of NAD+ synthesis.

### NMNAT3 alterations in Parkinson’s disease subject brain

PD is characterized by the progressive deconstruction of nigrostriatal pathway connectivity that occurs prior to degenerative cell loss (54). The affected striatal areas including the caudate nucleus are progressively depleted of dopamine and in turn neuronal activity (55), leading to dying back-like neurodegeneration (5). There is substantial clinical and experimental evidence that accumulation and aggregation of α-syn is one of the earliest triggers of disease onset and progression (56,57). This is particularly evident for early onset PD caused by duplication or triplication of the *SNCA* locus encoding α-syn (37,38). Despite extensive research into α-syn-mediated pathogenesis, the initiating causes underlying synaptopathy and axonal deconstruction have remained elusive (5).

Our *post mortem* analysis identified changes in NMNAT3 protein levels in caudate nucleus but not the cerebellum of PD patients. The detected changes were highly specific and significant as neither NMNAT1 nor NMNAT2 analysis revealed detectable alterations, suggesting that NMNAT3 dysfunction might be directly related to α-syn-mediated pathology. NMNATs are key players in NAD+ metabolism by catalyzing the chemical reaction of ATP with nicotinamide mononucleotide (NMN) into diphosphate and NAD+ (12). Dysfunction of NMNATs has been implicated in a multitude of diseases (12), including age-related neurodegeneration (13,18). So far, however, no study has reported a link between NMNAT3 and PD, which our findings provide for the first time.

The expression of NMNAT3 was approximately 50% lower in the cerebellum than that observed in the caudate nucleus. Regional variation in protein levels has been observed in several studies. For example, we have previously shown that the level of nicotinamide *N*-methyltransferase is lower in the cerebellum than the caudate nucleus (31) and higher than in the medial temporal lobe (58). A recent transcriptome analysis revealed that the expressions of 214 proteins are at least 4-fold higher in the cerebellum compared to all other brain regions (59). It is likely that the reason for the difference in NMNAT3 protein levels in the cerebellum and caudate is due to its relative importance in each region. It is also possible that it reflects a combination of different levels of NMNAT3 protein in neuronal and glial cells, and differing proportions of cell types in each region. It should be noted, however, that it is the relative change in expression between NDC and PD subjects which is key, for which we have previously shown the cerebellum to be a suitable region of choice as a control (58).

In support of this notion, the observed changes in NMNAT3 protein levels in the caudate nucleus were also detectable in the SN, as assessed by colocalization of NMNAT3 with both GFAP and TH using quantitative confocal microscopy. Our analysis revealed that the degree of colocalization between NMNAT3 and TH in DA neurones, but not with GFAP in glial cells, was significantly reduced compared to NDC subjects. These data suggest that neuronal levels of NMNAT3 protein are dysregulated not only in the caudate nucleus but also in the SN, indicating that altered NMNAT3 levels characterize PD.

### Alpha-synuclein causes alterations in NMNAT3 protein expression levels and neurite pathology

Our results reported here establish an inverse correlation between increased levels of monomeric α-syn and decreased NMNAT3 protein expression in PD caudate nucleus, suggesting a potential causal connection to α-syn-mediated synaptopathy and axonal deconstruction. In support of this hypothesis, ectopic expression of α-syn in differentiated DA neurone-like SH-SY5Y cells caused a severe reduction (70%) of NMNAT3 protein levels together with a concomitant loss of neurites. The observed effects were due to monomeric α-syn as evidenced by the lack of aggregated forms of α-syn in our SH-SY5Y model when compared to the expression of EGFP alone in SH-SY5Y^MOCK^ cells. Instead, our immunolocalization data are indicative of a direct interaction between α-syn and NMNAT3, which could result in enhanced degradation, and thus decreased levels, of NMNAT3 protein. Elucidating the exact mechanism is beyond the scope of this study and is something we will investigate further. While the exact mechanisms underlying these phenotypes remain to be established, our experiments suggest a direct link between PD-related α-syn pathology and neurite depletion that are associated with altered levels of NMNAT3 expression levels.

Earlier reports revealed the ability of NMNAT3 in preventing axonal degeneration where its ectopic expression delayed degeneration of transected sciatic nerves of mice, an effect not reproduced by NMNAT1 expression (22). NMNAT3 expression in dorsal root ganglia neurones also decreased axonal reactive oxygen species levels and protected against the toxicity of both rotenone and vincristine (23), and NMNAT3 was also able to protect axons of retinal ganglion RGC-5 cells (60). These data, together with our findings, indicate a neuroprotective role of NMNAT3 that might be diminished in PD due to decreased levels of NMNAT3 protein, as reported here both in patient’s brain and upon disease-related expression of α-syn in a cellular model of PD.

In contrast to NMNAT3, we did not observe significant alterations in the expression level of the pro-degenerative protein SARM1. A significant body of evidence has shown that SARM1 activation is integral to axonal degeneration by degrading NAD+ (15,61,62). Despite many such studies and reviews associating the SARM1 axonal degeneration pathway with PD (63), none have yet directly shown a dysfunction in SARM1 activity or its altered expression in a cohort of PD patients. A recent study reported increased levels of phosphorylated SARM1 in neuronal cells isolated from a single PD patient (64). Phosphorylation of SARM1 results in the activation of its NADase activity, thus suggesting that the cleavage of NAD+ by SARM1 is increased in this patient. It remains to be shown whether SARM1 phosphorylation is also altered in the PD subject cohort used in our study. Instead, our findings suggest that neither SARM1 nor NMNAT1 or NMNAT2 and thus Wallerian-like axonal degeneration (63,65) are involved in PD pathogenesis in the caudate nucleus of patient’s brain. Rather, our data indicate that accumulating α-syn and the resulting decrease in NMNAT3 protein levels trigger a yet to be identified pathogenic pathway affecting neurite integrity prior to the progressive deconstruction of neuronal connections and the subsequent degenerative cell loss seen in PD.

### Targeting NAD+ biosynthesis in *α*-synuclein pathology and Parkinson’s disease

Several lines of evidence suggest that mitochondrial dysfunction and altered NAD+ metabolism might be causally related to PD (12,13,66). NMNAT3 is expressed in mitochondria, where it regulates the provision of NAD+ for ATP energy production (19). Reduced ATP production in synaptic mitochondria has been shown to result in axonal death (67). Administration of nicotinamide and in turn increasing levels of NAD+ ameliorated mitochondrial defects and neurodegeneration in *Drosophila* models of PD with PINK1 mutations (68). A clinical case study revealed that nicotinic acid administration to a PD patient improved motor symptoms including rigidity and bradykinesia (69).

Based on these earlier findings, it is reasonable to suggest that a likely consequence of the observed reduction in NMNAT3 levels both in PD subject brain and in human cells with ectopic expression of α-syn, may in turn cause a reduction in mitochondrial NAD+ synthesis. As a result, the activity of NAD+-dependent mitochondrial pathways such as sirtuin-3 would also be reduced, thereby affecting downstream pathways such as the tricarboxylic acid cycle and β-oxidation along with reactive oxygen species generation (70–72). In this manner, NAD+ levels regulate sirtuin-mediated neurotrophic effects such as mitochondrial biogenesis, neurone function and plasticity (12,73,74). In support of this, reduced NAD+ levels and sirtuin-3 activity have been pathologically linked with the onset of PD (75). Second, reduced NAD+ synthesis may lead to reduced ATP synthesis. Third, NAD+ regulates calcium signaling *via* the production of cyclic-ADP ribose and 2"-O-cyclic-ADP-D-ribose (12,76). As a result, these likely phenotypes would converge onto and impair the structural integrity and function of neuronal connections including axons and synapses, as seen in PD (5).

A further consequence of the downregulation of NMNAT3 protein expression is an increase in the concentration of the pro-axonopathic molecule NMN. NMN is produced from its precursor, nicotinamide, by NAMPT (77). NAMPT is an essential enzyme catalyzing the first step in the biosynthesis of NAD+ from nicotinamide. In our *in vitro* experiments, we used the non-competitive inhibitor of NAMPT, FK866, which was able to rescue neurite pathology caused by ectopic α-syn expression in DA neurone-like SH-SY5Y cells.

FK866 is the most effective NAMPT inhibitor developed to date (78) and is used extensively in cancer research to target NAD+ synthesis and prevent cancer cell proliferation (79,80). The ability of FK866 to reverse the effects of α-syn expression in our *in vitro* model is in accord with previous studies which have shown corresponding effects. FK866 in combination with nicotinic acid riboside protects neurons from chemotherapy-induced axonal degeneration by bypassing NMN production (81). FK866 also prevents axonopathy in mouse models of spinal cord injury (82). The ability of FK866 to elicit a moderate increase the number of neurites in SH-SY5Y^MOCK^ cells most likely arises from a decrease in the cellular concentration of NMN. NMN is a prodegenerative molecule whose accumulation precedes axonopathy (83). SH-SY5Y^MOCK^ cells will contain NMN, which in the presence of FK866 may reduce sufficiently to elicit a moderate increase in neurite number. This is supported by our previous studies of the expression of nicotinamide *N*-methyltransferase in SH-SY5Y cells, which in tandem with decreased intracellular NAD+ levels and NMN synthesis also increased the number of neurites per cell (84). This also explains why FK866 induced a much more marked increase in neurite number in SH-SY5Y^WT^, as these cells would contain significantly higher NMN levels due to decreased NMNAT3 expression.

Although several pharmacological approaches are currently being developed to target α-syn in PD, to our knowledge, this is the first study to demonstrate a pharmacological intervention which can reverse α-syn-induced neurite pathology *in vitro* or *in vivo*. The ability of FK866 to rescue α-syn-mediated neurite pathology in our cell model, if replicated in *in vivo* models, would suggest that targeting the NAD+ biosynthetic pathway, and NAMPT in particular, may be a viable therapeutic target for preventing, and potentially reversing, α-syn-mediated degeneration of neuronal connections seen in PD. In patients already demonstrating symptoms, such a therapeutic strategy would potentially slow down, or even mitigate, the progression of disease, resulting in significant improvements in patient quality of life.

## Materials and Methods

All materials were obtained from Sigma-Aldrich (Poole, UK) unless otherwise stated and were of the highest grade available.

### Human tissue

Flash-frozen *post mortem* tissue from the caudate nucleus and cerebella of 19 NDC and 19 idiopathic PD patients were obtained from the Parkinson’s UK Brain Bank, Imperial College, London, UK (average age ± SD, NDC: 79.4 ± 8.3 years, range 65–93 years; PD: 78.5 ± 5.7 years, range 65–87 years). *Post mortem* interval for all samples was below 48 hours (average ± SD 18.1 ± 1.3 hours, range 3–48 hours). Ethical permission for their use in this study was obtained from the Wales Research Ethics Committee (Ref No. 07/MRE09/72). Formalin-fixed, paraffin-embedded (FFPE) *post mortem* tissue from the SN and cerebella of four NDC and four idiopathic PD patients were obtained from the Queen Square Brain Bank for Neurological Research, University College London, London, UK (NDC: 74.5 ± 13.4 years, range 56–88, 2 male, 2 female; PD: 75.3 ± 9.1 years, range 67–88, 2 male, 2 female). Ethical permission for their use in this study was obtained from the National Health Service Research Authority NRES Committee London – Central (REC# 08/H0718/54 + 5). Patients with a history of cancer were excluded from both cohorts due to the changes in the expression levels of enzymes of the NAD+ biosynthetic pathway in this disease (77). Full clinical information for all participants, including genotypes for PD-related genes, obtained from the Parkinson's UK Brain Bank are summarized in Table 1. The *SNCA* genotype of patients has not been determined by the Parkinson's UK Brain Bank and is thus unknown.

### Quantitative western blotting

Flash-frozen tissues were prepared for sodium dodecylsulphate/polyacrylamide gel electrophoresis/western blotting as previously described using radioimmunoprecipitation assay buffer (31,85). Protein expression was detected using combinations of primary and secondary antibodies as summarized in Table 3. Bands were visualized using electrochemiluminescence detection and quantified using FIJI Image-J v1.53a. Protein molecular weights were estimated using a calibration curve constructed using streptavidin-tagged Precision Plus Protein Markers (Bio-Rad, Hemel Hempstead, UK). Streptavidin-tagged markers were processed separately, yet in parallel, with the rest of the blot to prevent oversaturation of the electrochemical signal obtained from proteins detected in cell samples. Protein molecular weights were expressed as kDa; we have previously shown that such estimates have an error of 9.3% (86). Representative whole western blots for all target proteins are shown in Supplementary Material, Figure S2. Protein levels were normalized using the housekeeping protein β-tubulin and expressed as normalized protein expression. Protein expression for each group was calculated and expressed as normalized tubulin ratio, with error bars quoted as ±SEM for results obtained using patient tissue and as ± SD for results obtained using *in vitro* cell models.

**Table 3:** Primary and secondary antibodies used in the study

**Protein**	**Primary antibody**		**Secondary antibody** ^**a**^	
	**Dilution**	**Product code**	**Dilution**	**Product code**
NMNAT1	1:1000	ab10517 (Abcam)	1:2000	A0545 (Sigma)
NMNAT2	1:500	sc-515 206 (Santa Cruz Biotechnology)	1:2000	A0545
NMNAT3 WB Confocal microscopy	1:1000 1:50	ab71904 (Abcam) ab71904 (Abcam)	1:2000 1:200	A0545 AlexaFluor™ 594 A-21201 (Molecular Probes)
SARM1	1:500	HPA024359 (Atlas Antibodies)	1:2000	A9917 (Sigma)
TH	1:1000	2792S (Cell Signalling Technology)	1:2000	A9917
α-Syn WB Aggregates	1:1000 1:1000	26 475 (Cell Signalling Technology) 864 901 (BioLegend)	1:2000 1:2000	A0545 A9917
NeuN	1:2000	MAB377 (Merck Millipore)	1:2000	A0545
β-Tubulin	1:1000	ab180207 (Abcam)	1:5000	A4416 (Sigma)

^a^All secondary antibodies used for western blot were anti-IgG conjugated to horseradish peroxidase. NMNAT, nicotinamide mononucleotide adenylyltransferase; SARM1, sterile alpha and TIR motif containing 1; TH, tyrosine hydroxylase; α-Syn, α-synuclein; NeuN, neuronal nuclear protein/FOX-3; WB, western blot.

### Double-label confocal microscopy

FFPE sections were prepared and proteins detected, using combinations of primary and secondary antibodies summarized in Table 3 as previously described (31). The sections were counterstained using 4′,6-diamidino-2-phenylindole (DAPI) and mounted in ProLong Gold™ antifade mountant (Life Technologies, UK). Images were captured using a Nikon A1R inverted confocal microscope, using a x40 objective lens, as z-stacks at a resolution of 1024×1024 pixels per inch. Stacks were subsequently rendered into maximum projection images and saved as .tiff files which were used as inputs for subsequent colocalization analysis. Colocalization was estimated using the EzColocalization plug-in v1.1.3 in FIJI Image-J v1.53a (58). Correlation between NMNAT3 with both TH and GFAP staining was estimated using TOS, using Costes’ thresholds, and expressed as TOS ± SD. Overlap between the expression of each protein was estimated using Mander’s colocalization coefficients using Costes’ thresholds and were expressed as percentage ± SD.

### Culture of SH-SY5Y human neuroblastoma α-synuclein *in vitro* models

SH-SY5Y^MOCK^ and SH-SY5Y^WT^ cells (a kind gift from Dr Sebastien Paillusson, King’s College London) were cultured in 1:1 Dulbecco’s modification of Eagle:Ham’s F12 medium supplemented with fetal calf serum (10% v/v), 2 mM glutamine (Life Techonologies, Paisley, UK), sodium pyruvate (1% v/v), non-essential amino acids (1% v/v), 100 U/ml penicillin, 100 μg/ml streptomycin, and 500 μg/ml geneticin (Life Technologies, Paisley, UK). Cells of passage number 8–25 were used in experiments. Cells were terminally differentiated into DA neurones using 10 μM retinoic acid as previously described (41,43), which was confirmed using western blotting detection of the post-mitotic marker NeuN and the DA marker TH using antibody combinations described in Table 3. α-syn and NMNAT3 protein levels were determined by quantitative western blotting detection using combinations of primary and secondary antibodies described in Table 3. The formation of soluble aggregates of α-syn was assessed using of an antibody raised against aggregated forms of α-syn described in Table 3.

### Dual-label immunocytofluorescence

Cells were seeded onto poly-lysine-coated microscope slides at a density of 400 000 cells/ml and allowed to settle overnight, after which they were terminally differentiated as described above. Cells were fixed using 4% paraformaldehyde in phosphate-buffered saline, after which NMNAT3 was detected using a combination of primary and secondary antibodies outlined in Table 3. Cells were counterstained using DAPI, mounted in ProLong Gold™ mounting medium and visualized using a Nikon A1 inverted confocal microscope, using a x40 objective lens. Cells were also imaged using a x40 objective with 2-fold software zoom. All images were subsequently rendered into maximum projection images.

### Quantitative polymerase chain reaction

Cells were differentiated as described above, after which mRNA was isolated using the peqGOLD Total RNA Kit (VWR, Darmstadt, Germany). After elution, a DNase step was performed in solution to remove residual genomic DNA. Total isolated RNA was diluted to 100 ng/μL in nuclease-free water. The integrity of isolated RNA was assessed by running approximately 200 ng on a 1% agarose gel. Reverse transcription was performed on 1 μg of purified RNA per sample using the Ultrascript 2.0 cDNA synthesis kit (PCR Biosystems, London, UK). Quantitative PCR was performed on 4-fold diluted cDNAs using the qPCRBIO SyGreen Mix with Fluorescein (PCR Biosystems) in an IQ5 thermal cycler (BioRad, Hemel Hempstead, UK) using NMNAT3 primers outlined in Table 4. β-Actin (Table 4) was used as the endogenous control. Primer efficiencies were assessed using 10-fold dilutions of pooled cDNA and were found to be 102% for β-actin and 105% for NMNAT3. Thermal cycling profiles for all primer pairs followed those recommended for the qPCR kit. Specificity of all reactions were determined using melt curve analysis. Non-template (RNA) controls were included to assess for DNA contamination. Abundance of NMNAT3 transcript was calculated using the Pfaffl method (87) and expressed as the mean expression ratio ± SD.

**Table 4:** Primers for the analysis of messenger RNA expression using real-time quantitative polymerase chain reaction

**Gene name**	**Accession number**	**Forward/reverse**	**Primer sequence**	***T***_**m**_ **(°C)**
*NMNAT3*	NM_001320510	Forward	CACAACATTCACCTGGCCAA	56
		Reverse	GGTACTTTACGCTCTGCCCT	
*ACTB*	NM_001101.3	Forward	CCAACCGCGAGAAGATGA	59
		Reverse	CCAGAGGCGTCAGGGATAG	

*NMNAT3*: nicotinamide mononucleotide adenylyltransferase3; *ACTB*: β-actin; T_m_: annealing temperature

### Effect of FK866 upon cell morphology

Neurone morphology was assessed as previously described (88). Briefly, SH-SY5Y^MOCK^ and SH-SY5Y^WT^ cells were seeded at a concentration of 100 000 cells/ml and allowed to settle overnight. Cells were imaged under phase-contrast microscopy using an Axiovert C40 microscope. Cells were subsequently incubated with 50 nM FK866 for 24 hours and then imaged again. Neurite length was quantified using the NeuronJ v.1.4.3 plugin for FIJI-J v.1.53a (89). Only neurites greater than 10 μm were considered as true neurite processes and as such counted (88). Neurite length was expressed as mean length in μm ± SD. Median and quartiles were also calculated. The total number of neurites counted were 195 for SH-SY5Y^MOCK^, 489 for SH-SY5Y^MOCK^ incubated with FK866, 47 for SH-SY5Y^WT^ and 226 for SH-SY5Y^WT^ incubated with FK866. The average number of neurites per cell was calculated and expressed as neurites per cell ± SD. The total number of cells imaged were 240 for SH-SY5Y^MOCK^, 366 for SH-SY5Y^MOCK^ incubated with FK866, 332 for SH-SY5Y^WT^ and 336 for SH-SY5Y^WT^ incubated with FK866.

### Statistical analysis

All statistical analyses were performed using Prism v8.3 (GraphPad, San Diego, USA). Comparisons of protein expression levels between (i) PD and NDC subjects, (ii) PD subjects with and without dementia, (iii) PD subjects with and without AD pathology, (iv) PD subjects with Tau score < 2 and Tau score = 2, and (v) SH-SY5Y^MOCK^ and SH-SY5Y^WT^ were performed using Student’s *t*-test with Welch correction. Comparison of protein expression levels between NDC and PD subjects without dementia and with dementia was performed using one-way analysis of variance (ANOVA) with Dunnett’s T3 *post hoc* multiple comparisons. Correlations between protein expression levels and (i) potential confounding factors as shown in Table 1 and (ii) soluble α-syn protein levels were performed using Spearman’s correlation coefficient analysis. Comparison of TOS, Mander’s coefficients M1 and M2, and NMNAT3 mRNA levels were performed using Student’s *t*-test with Welch correction. QQ plots of residuals were used to test whether average neurite length and average number of neurites per cell followed a normal distribution. Consequently, comparisons of the effects of α-syn protein expression level and incubation with FK866 upon neurite length were performed using non-parametric one-way ANOVA with Kruskal–Wallis multiple comparison tests. Also, comparisons of the effects of α-syn protein expression and incubation with FK866 upon the average number of neurites per cell were performed using parametric one-way ANOVA with Tukey’s multiple comparison tests. In all cases, *P* < 0.05 was considered as significant.

## Supplementary Material

HMG-2021-CE-00484_Parsons_Supplementary_information_ddac077
