# Supplementary material for: Alpha-synucleinopathy reduces NMNAT3 protein levels and neurite formation that can be rescued by targeting the NAD+ pathway
Source: Hum Mol Genet. 2022 Apr 9;31(17):2918–33. doi: 10.1093/hmg/ddac077 (PMC9433734; doi:10.1093/hmg/ddac077)
Supplement: HMG-2021-CE-00484_Parsons_Supplementary_information_ddac077 [file hmg-2021-ce-00484_parsons_supplementary_information_ddac077.docx]

**Supplementary information for Parsons et al. “Alpha-synuclein reduces NNMT protein levels and neurite formation that can be rescued by targeting the NAD+ pathway”**

This word document comprises 4 tables and two figures. Table S1 comprises the comparison of the levels of NMNATs 1 & 2 plus SARM1 in patient *post mortem* tissue, with Figure S1 showing the individual data points. Tables 2, 3 and 4 comprise the comparison of the effects of clinicopathological confounding factors upon NMNAT3 protein levels in patient *post mortem* tissue. Figure S2 shows full-length Western blot images for all target proteins investigated.

*Abbreviations used:* 1 – 8 = patient sample lanes; D = retinoic acid-differentiated SH-SY5Y^MOCK^ cells; kDa = kilodaltons; M = molecular weight markers; n/a: not applicable; NDC: non-disease controls; NeuN = neuronal nuclear protein/Fox-3; NMNAT1, -2 and -3: nicotinamide mononucleotide adenylyltransferase-1, -2 and -3 respectively; PD: Parkinson’s disease; SARM1: SARM1 = sterile alpha and TIR motif containing 1; SEM: standard error of the mean; TH = tyrosine hydroxylase; U = undifferentiated SH-SY5Y^MOCK^ cells

|  | **Caudate nucleus** | | | **Cerebellum** | | |
| --- | --- | --- | --- | --- | --- | --- |
|  | **NDC** | **PD** | ***P* - value** | **NDC** | **PD** | ***P* - value** |
| **NMNAT1** | 0.74 ± 0.15 | 0.58 ± 0.08 | 0.34 | 0.89 ± 0.27 | 0.67 ± 0.15 | 0.48 |
| **NMNAT2** | 0.5 ± 0.1 | 0.39 ± 0.11 | 0.46 | 0.74 ± 0.28 | 0.95 ± 0.28 | 0.61 |
| **SARM1** | 0.68 ± 0.11 | 0.65 ± 0.09 | 0.83 | 0.68 ± 0.15 | 0.74 ± 0.16 | 0.8 |

**Table S1. Comparison of tubulin-corrected expression of NMNAT1, NMNAT2 and SARM1 in NDC and PD subjects.** Results are expressed as NMNAT3:tubulin ratio ± SEM (*n* = 19 for both groups). None of the values are significant (*t*-test with Welch correction)





**Figure S1. Quantitative analysis of target proteins in the caudate nucleus and cerebellum of non-disease control and Parkinson’s disease subjects**

| **Tau score <2** | **Tau score = 2** | ***P* - value** |
| --- | --- | --- |
| 0.43 ± 0.18 | 0.39 ± 0.06 | 0.83 |

**Table S2. Effect of Tau pathology on NMNAT3 expression in PD subjects.** Results are expressed as NMNAT3:tubulin ratio ± SEM. Statistical analysis comprised Student’s *t*-test with Welch correction (n = 7 and 12 for Tau score <2 and Tau score = 2 respectively)

| **No Alzheimer’s pathology** | **Alzheimer’s pathology present** | ***P* – value** |
| --- | --- | --- |
| 0.36 ± 0.08 | 0.43 ± 0.13 | 0.66 |

**Table S3. Effect of the presence of Alzheimer’s-associated pathology upon NMNAT3 expression in PD subjects.** Results are expressed as NMNAT3:tubulin ratio ± SEM. Statistical analysis comprised Student’s *t*-test with Welch correction (*n* = 9 and 10 for no pathology and pathology present respectively).

|  | **PD subjects** | | **Whole cohort** | |
| --- | --- | --- | --- | --- |
|  | r | ***P* – value** | **r** | ***P* – value** |
| **Age of death** | 0.2 | 0.42 | -0.12 | 0.47 |
| ***Post mortem* interval** | -0.28 | 0.27 | -0.03 | 0.84 |
| **RIN** | -0.04 | 0.86 | -0.26 | 0.13 |
| **Age of disease onset** | -0.05 | 0.84 | n/a | n/a |
| **Disease duration** | 0.26 | 0.28 | n/a | n/a |
| **Age of onset of dementia** | 0.3 | 0.37 | n/a | n/a |
| **Duration of dementia** | -0.49 | 0.13 | n/a | n/a |

**Table S4. Correlation between pathological data and NMNAT3 expression.** Statistical analysis comprised Pearson r correlation coefficient analysis. For Parkinson’s disease and whole cohort analyses, *n* = 19 and 37 respectively


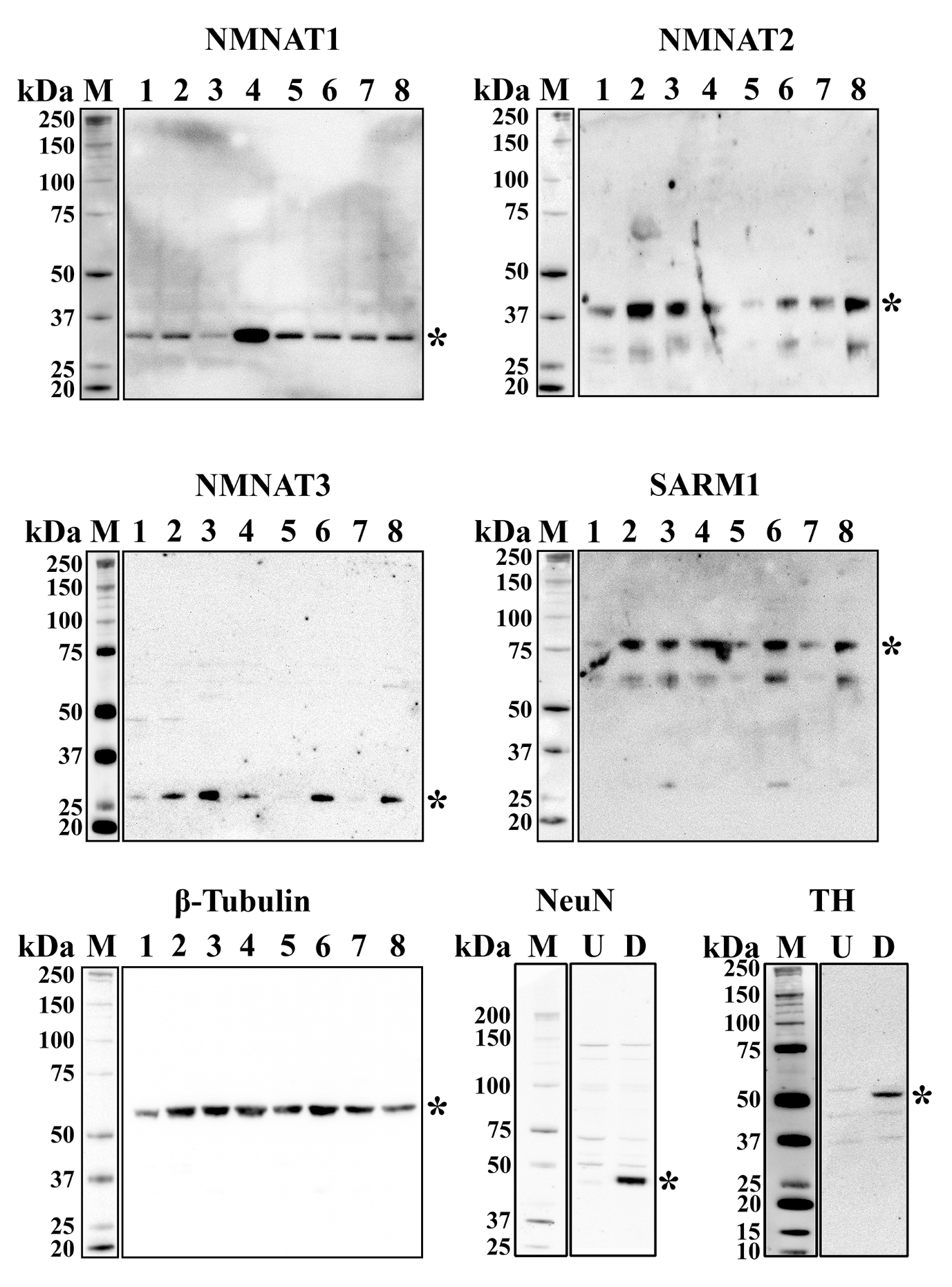


**Figure S2. Representative whole Western blots of target proteins used in this study.** Molecular weight markers were electrophoresed on the same gel and were cut from the nitrocellulose membrane prior to ECL development to prevent potential saturation of target protein signal. Images were digitally reformed, with frames showing the separation of markers from target protein images. All target protein molecular weights were calculated using a calibration line derived from its associated molecular weight maker lane and expressed as kDa. Asterix denotes the target protein of interest and corresponds to the known molecular weight of the target protein. Other bands in common in each lane represent non-specific binding and were not analysed
